# Bromodomain Inhibitors as Therapeutics for Herpesvirus-Related Disease: All BETs Are Off?

**DOI:** 10.3389/fcimb.2020.00329

**Published:** 2020-07-02

**Authors:** Ian J. Groves, John H. Sinclair, Mark R. Wills

**Affiliations:** Department of Medicine, Addenbrooke's Hospital, University of Cambridge, Cambridge, United Kingdom

**Keywords:** herpesvirus, BET bromodomain inhibitor, epigenetics, latency, reactivation, therapy

## Abstract

Although the ubiquitous human herpesviruses (HHVs) are rarely associated with serious disease of the healthy host, primary infection and reactivation in immunocompromised individuals can lead to significant morbidity and, in some cases, mortality. Effective drugs are available for clinical treatment, however resistance is on the rise such that new anti-viral targets, as well as novel clinical treatment strategies, are required. A promising area of development and pre-clinical research is that of inhibitors of epigenetic modifying proteins that control both cellular functions and the viral life cycle. Here, we briefly outline the interaction of the host bromo- and extra-terminal domain (BET) proteins during different stages of the HHVs' life cycles while giving a full overview of the published work using BET bromodomain inhibitors (BRDis) during HHV infections. Furthermore, we provide evidence that small molecule inhibitors targeting the host BET proteins, and BRD4 in particular, have the potential for therapeutic intervention of HHV-associated disease.

## Introduction

The mammalian *herpesviridae* comprises three sub-families (alpha, beta, and gamma) that likely arose around 200 million years ago (McGeoch et al., 1995; Mettenleiter et al., 2008). As such, human herpesviruses (HHVs) have co-evolved with their host and primary infection is usually associated with few minor non-life threatening symptoms, although some ailments are associated with discomfort or more chronic manifestations (e.g., cold sores, genital lesions, mononucleosis; summarized in Table 1) (Arvin et al., 2007). With well over 90% of the world population infected with at least one HHV, these human pathogens have developed a particular method of maintaining their presence in the infected host. Common to all herpesviruses, after primary infection, they establish a so-called latent infection where virus genomes are carried in specific sites in the host with the absence of production of infectious virions. The cellular tropism of each HHV sub-family determines where virus lies latently for the lifetime of the host but, importantly, where it is routinely able to undergo reactivation from, allowing the production of new infectious virions and transmission to a new host.

**Table 1:** Characteristics of human herpesvirus (HHV) infections.

**HHV official nomenclature**	**Common name**	**HHV type**	**Primary pathology**	**Further clinical conditions**	**Primary target cells**	**Sites of latency**	Common therapy^*^
HHV-1	Herpes simplex virus 1 (HSV-1)	Alpha	Cold sores	Skin lesions, keratitis, encephalitis, meningitis	Mucosal and epithelial cells	Neurons	ACV, FAM, VAL, PCV
HHV-2	Herpes simplex virus 2 (HSV-2)	Alpha	Genital lesions, cold sores	Skin lesions, keratitis, encephalitis, meningitis	Mucosal and epithelial cells	Neurons	ACV, FAM, VAL
HHV-3	Varicella zoster virus (VZV)	Alpha	Chicken pox	Herpes zoster, shingles	Mucosal and epithelial cells	Neurons	ACV
HHV-4	Epstein-Barr virus (EBV)	Gamma	Mononucleosis/ glandular fever	Lymphoma, nasopharyngeal carcinoma (NPC), T/NK cell and gastric cancers	Epithelial and B cells	Memory B cells	Steroids
HHV-5	Human cytomegalovirus (HCMV)	Beta	Mononucleosis, mental retardation (congenital infection)	Periodontitis, retinitis, pneumonitis, hepatitis, nephritis	Epithelial and myeloid cells	Myeloid cells	GCV, FOS, LET
HHV-6A &−6B	Roseola virus (HHV-6)	Beta	Exanthema subitum (roseola), rash		T cells	Leukocytes	None
HHV-7	Roseola virus (HHV-7)	Beta	Exanthema subitum (roseola), rash		T cells	T cells	None
HHV-8	Kaposi's sarcoma-associated herpesvirus (KSHV)	Gamma	Fever, rash	Kaposi's sarcoma (KS), primary effusion lymphoma (PEL) and multicentric Castleman's Disease	Epithelial cells and lymphocytes	B cells	Radiation, cytotoxic drugs, IFN-α, GCV

* *Treatments updated from Coen and Schaffer (2003). ACV, acyclovir; FAM, famciclovir; FOS, foscarnet; GCV, ganciclovir; IFN-α, interferon-α; LET, letermovir; PCV, penciclovir; VAL, valacylovir*.

Significantly, it is reactivation of HHVs in an immunocompromised individual that commonly leads to life threatening illness; for instance, patients with acquired immunodeficiency syndrome (AIDS) from human immunodeficiency virus (HIV) infection or those undergoing transplantation operations and who are immunosuppressed (Arvin et al., 2007). Various anti-viral drugs can be employed successfully both prophylactically and pre-emptively. Current herpesvirus anti-virals used within the clinic are able to impair DNA/RNA synthesis [e.g., (val)acyclovir, famciclovir, (val)ganciclovir, cidofovir]. However, these nucleoside analogs only target lytically infected cells, suffer from poor bioavailability and can have profound toxic side effects (Field and Vere Hodge, 2013). Further compounds have been purposed experimentally against other stages of the HHV lifecycle, including virus-cell binding and entry, as well as virion assembly and egress (e.g., maribavir) (Coen and Schaffer, 2003). However, even contemporary drugs, such as letermovir that inhibits the DNA terminase complex during human cytomegalovirus (HCMV) replication, have rising examples of resistance (Popping et al., 2019; Douglas et al., 2020). Therefore, with a lack of tractable vaccinations for the majority of HHVs (with the exception of varicella zoster virus (VZV) (Field and Vere Hodge, 2013; Plotkin, 2020), in part due to their ability to both hide latently and actively evade the immune system, one avenue during iatrogenic-induced reactivation might be to reduce the latent cell reservoir prior to clinical treatment (Krishna et al., 2019). This is no more relevant than with HCMV reactivation during solid organ or haematopoietic stem cell transplantation (SOT or HSCT, respectively) where the prospect of a “shock and kill” approach would be advantageous (Poole et al., 2014; Wills et al., 2015). Here, an agent such as a small molecule inhibitor targeting host proteins involved in the maintenance of latency allows reactivation of the virus in a relatively healthy host such that the immune system is able to purge the latently infected reservoir (Nehme et al., 2019). Unfortunately, despite some success with histone deacetylase inhibitors (HDACis), this regimen is proving inefficient with HIV treatment (Ait-Ammar et al., 2019), therefore further targets must be established.

**Figure 1:**
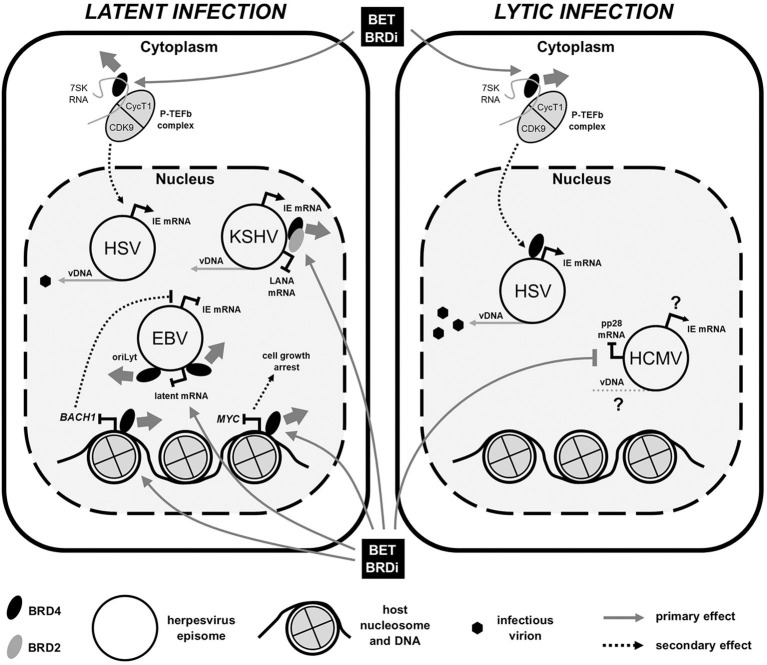
BET bromodomain inhibitor (BRDi) effects on human herpesvirus (HHV) infection. Diagram summarizes the published effects of BET BRDi treatment on viral gene expression of both HHV latent **(Left)** and lytic **(Right)** infections. Briefly, BET BRDi treatment of cells causes release of BRD4 (thick gray arrow) from DNA, repressing host transcription, reactivation of EBV and cell growth. Dissociation of BRD4 (and BRD2) from virus genomes can both inhibit DNA replication (EBV) and reactivate KSHV, whilst release and redistribution of P-TEFb causes both reactivation and lytic augmentation of HSV. Effects of BET BRDi on HCMV are largely unknown. Elements of figure not to scale. IE, immediate early; mRNA, messenger RNA; vDNA, viral DNA.

Cellular transcription factors are routinely hijacked and employed by viral pathogens throughout the course of the viral lifecycle and one group of these is the bromo- and extra-terminal domain (BET) proteins. The BET protein group comprises BRD2, BRD3, BRD4, and the testis-specific BRDT (Zaware and Zhou, 2019). In addition to an extra-terminal domain, human BET proteins contain two BRDs (BD1 and BD2) through which they can interact with acetylated proteins (Filippakopoulos et al., 2012). Through their association via both BRD and ET domains, BET proteins are known to be involved in many cellular processes, including control of transcription, regulation of DNA replication and cell cycle through “reading” of cellular histone lysine acetylation marks and acting as a scaffold for transcription factor recruitment. Due to their association with transcriptional dysregulation in various lymphomas and leukaemias, BET proteins, and more specifically BRD4, have become important targets for cancer treatment and, as such, many small molecule inhibitors are now available (Bhattacharya et al., 2018; Cochran et al., 2019). Interestingly, BRD4 inhibitors either alone or in combination have been shown to cause reactivation of HIV and have shown some promise in “shock and kill” pre-clinical studies (Halper-Stromberg et al., 2014; Cary et al., 2016; Nehme et al., 2019). Here, we briefly review the known interactions between herpesvirus lifecycles and the host BET proteins and also the effects that BRD inhibitors (BRDi) have been found to have at the molecular level (Figure 1) and within the clinical setting toward whether BRDi could be used routinely for herpesvirus-related disease.

## BET Bromodomain Inhibition and Gammaherpesviruses

The initial specificity of BET BRDis allowed targeting of the ability of BET proteins to bind to acetyled lysine marks on histones found at active areas of the host genome, thereby inhibiting the cellular transcriptional dysregulation occurring in malignancies such as sarcoma and leukemia (Filippakopoulos et al., 2010; Dawson et al., 2011). Due to the association of gammaherpesviruses with various cancers, it is not surprising that the majority of virus publications including the use of BET BRDis involve infection with these herpesviruses in particular. After primary infection, the two human gammaherpesviruses Epstein-Barr virus (EBV) and Kaposi's sarcoma-associated herpesvirus (KSHV) can reside latently in B lymphocytes. While both viruses are associated with various lymphomas, EBV is also an etiologic agent of gastric cancers and nasopharyngeal carcinomas (NPCs) (Farrell, 2019). EBV is unique in the human herpesviruses by its use of distinct latency transcription profiles in which a few select viral gene products including the latent membrane proteins (LMPs) and EBV nuclear antigens (EBNAs), driven from separate promoters on the virus genome, are expressed in a B cell differentiation-specific manner. These different latency transcription programs (Lat I-III) are known to be regulated, at least in part, through epigenetic modification to viral chromatin (Hammerschmidt, 2015). Application of the most widespread BET BRDi inhibitor, JQ1 (Filippakopoulos et al., 2010), to EBV-infected Burkitt lymphoma (BL) cell lines caused a decrease in transcription from the C promoter (Cp) (a necessary process in cellular immortalization) but, interestingly, not the LMP1 promoter (Palermo et al., 2011). Inhibition of BRD4 interaction through acetylated histones at Cp was shown to decrease the recruitment of the positive transcription elongation factor (P-TEFb) complex, composed of cyclin T1 (CycT1) and cyclin-dependent kinase 9 (CDK9), the latter which activates poised/paused RNA polymerase II (RNAPII) by phosphorylation of serine 2 on the C-terminal domain (CTD) (Chen et al., 2018). This JQ1-driven mechanism also inhibits the expression of host genes in EBV-infected cell lines, whereby transcription from virus-modulated host super-enhancers, such as that controlling MYC expression, is restricted. Here, MYC down-regulation led to growth inhibition of lymphoblastoid cell lines (LCLs) (Zhou et al., 2015), which was also consistent with treatment of NPC cell lines elsewhere (Li et al., 2018).

Most promising therapeutically is a report of BRDi allowing depletion of LCLs in an *ex vivo* co-culture setting. JQ1 treatment caused up-regulation of LMP1 and subsequent modulation of down-stream signaling pathways as well as increased MHC class I presentation, the latter likely allowing cytotoxic T cells to target once hidden cells (Smith et al., 2016). Indeed, BRDi (JQ1 and iBET-762, a benzodiazepine-based BET BRDi) (Nicodeme et al., 2010; Mirguet et al., 2013) treatment of a BL cell line with EBV in latency I (Q promoter driven transcription) added further evidence as to the possible safety of using these inhibitors; BRDi caused inhibition of host BACH1 expression, which is necessary to drive virus reactivation through the immediate early (IE) protein BZLF1 (Keck et al., 2017). Concomitant inhibition of BRD4 interaction with the EBV origin of lytic (oriLyt) replication caused complete prevention of EBV lytic cycle and, as such, would provide favorable conditions for treatment of EBV-associated malignancies.

Analogous to EBV, KSHV gene expression and latency is controlled by epigenetic modification (Chen et al., 2013) and, as such, BRDi have been trialed in pre-clinical studies of KSHV-associated malignancies. Either JQ1, iBET-151 or PFI-1 (a dihydroquinazoline-2-one BET BRDi) (Picaud et al., 2013) treatment of primary effusion lymphoma (PEL) cells has shown decreased cell and tumor growth rates, as well as increased survival, in PEL xenograft models (Tolani et al., 2014; Gopalakrishnan et al., 2016; Zhou et al., 2017). However, effects to PEL cell growth were predominantly caused by the down-regulation of MYC expression. KSHV reactivation, and subsequent oncolysis, was only initiated when cells were co-treated with JQ1 and an NF-κB-activating compound (PEP005) (Zhou et al., 2017). This is in contrast to reports elsewhere of KSHV reactivation with BET BRDi alone (Chen et al., 2017; Hopcraft et al., 2018), although the disparity in results may be due to the range of PEL cell and reporter lines used across all of these studies, some of which are also EBV-positive. In a sophisticated study, the Lieberman laboratory resolved the molecular mechanism of BET BRDi-induced KSHV reactivation; using JQ1, they showed that the three-dimensional (3D) looping conformation of the KSHV genome that maintains the expression of latent transcripts (Chen et al., 2013) was destabilized such that lytic expression ensued (Chen et al., 2017). The presence of both BRD2 and BRD4 proteins on the virus genome was demonstrated to stabilize transcription at the latency control region producing the latency-associated nuclear antigen (LANA), whilst interaction of BRD2/4 with LANA protein itself was seen at virus genome terminal repeats (TRs) regulating loop structure. With BET protein inhibition, LANA was released from the KSHV genome causing the shift in 3D conformation and virus reactivation (Chen et al., 2017).

As well as maintaining the latent expression profile of KSHV, the LANA protein also acts as a viral episome maintenance protein (EMP) tethering the extrachromosomal genomes to host chromosomes through interaction with host proteins. Human gammaherpesvirus EMPs appear to interact with at least BRD4 (De Leo et al., 2020) and, as such, could be targeted therapeutically with BET BRDi to restrict genome carriage. One study with racoon polyomavirus (RacPyV), a small DNA virus shown to tether its genome to the host chromosome with BRD4, showed promising results with JQ1-induced reductions in viral transcripts and genome copy number in neuroglial tumor cells (Church et al., 2016). However, given that BRDi treatment reactivates KSHV (Chen et al., 2017; Hopcraft et al., 2018) and BRD4 depletion in an EBV-infected NPC cell line didn't affect genome segregation (Lin et al., 2008), this might be a less tractable therapeutic angle with HHVs.

## BET Bromodomain Inhibition and Alphaherpesviruses

The human alphaherpesviruses are characterized by latent infection of cells of the nervous system from where they are able to cause repeated pathology. Herpes simplex virus-1 (HSV-1),−2, and VZV primarily infect cells of the mucosal epithelia before dissemination to sensory neurons of the peripheral nervous system (Arvin et al., 2007). VZV is named directly after the clinical symptoms associated with infection, varicella (chickenpox) and zoster (shingles), the latter being associated with virus reactivation. Both are normally self-limiting, with severe pathology usually only arising in the immunocompromised host (Steiner et al., 2007). VZV is the only herpesvirus for which an effective vaccine has been developed and, as such, has been available for immunization of children in the USA since 1995 (Field and Vere Hodge, 2013). No studies of VZV and BET proteins or their inhibitors have been reported.

In contrast to VZV, investigation into the effects of BET BRDi on HSV during lytic and latent infection has recently been reported. Due to the cellular tropism of the simplex viruses, reactivation events of HSV-1 and -2 can repeatedly cause epithelial lesions both orally (cold sores) and genitally, with occasional transfer to the central nervous system giving rise to encephalitis and systemic disease (Whitley and Baines, 2018). Like other herpesviruses, HSV gene expression during both lytic and latent infection is governed by epigenetic regulation (Kristie, 2015) and, thus, led to investigation of a number of BET family inhibitors. Employment of JQ1, as well as other BET BRDi (iBET-762, HMBA, and PFI-1), over two studies has shown increases in HSV transcript levels, protein production and virus output from lytic infection systems (Ren et al., 2016; Alfonso-Dunn et al., 2017). Indeed, JQ1 was also able to reactivate HSV-1 from a mouse sensory ganglia explant and *in vivo* mouse models (Alfonso-Dunn et al., 2017). Both studies showed the enhanced association of CDK9, the transcriptional activator of the P-TEFb complex, with HSV promoters driving virus transcription. The Li group provided evidence that P-TEFb was reallocated to virus promoters by BRD4 after JQ1 treatment, supported by experiments using small interfering RNAs (siRNA) targeting BRD4 to decrease virus transcription (Ren et al., 2016). However, the Kristie group showed association of P-TEFb after JQ1 treatment to be independent of BRD4 at the virus genome, with depletion of BRD4 with siRNA causing increases in HSV IE transcript levels (Alfonso-Dunn et al., 2017). The latter mechanism was determined to be caused by the release of P-TEFb from repressive 7SK snRNP complexes in the cytoplasm (Bartholomeeusen et al., 2012), comparable to HIV studies elsewhere (Biglione et al., 2007), with recruitment being through AFF4 and the super elongation complex (SEC) (Chen et al., 2018). Interestingly, use of RVX-208 which targets BD2 of BRD4, showed no effect on HSV replication, confirming that regardless of downstream mechanism, JQ1 interferes with BRD4 activity through interaction via BD1 (Ren et al., 2016).

Outside of human herpesviruses, a study on the swine alphaherpesvirus pseudorabies virus (PRV) has identified various means by which BRD4 inhibition can affect lytic virus infection. Use of three different compounds (JQ1, OTX015, and iBET-151), alongside confirmatory data with siRNA use, has recently shown that inhibition of BRD4, due to its importance in maintenance of higher-order chromatin structure (Wang et al., 2012), can lead to activation of the DNA damage response by chromatin de-compaction (Wang et al., 2020). In concert with induction of interferon-stimulated genes (ISGs), including IFN-β, DNA damage triggers activation of cGAS-mediated innate immunity and is able to attenuate attachment of the virus to the surface of BRDi-treated cells (Wang et al., 2020). In the absence of any effects to virus transcription, this BRDi-driven induction of an anti-viral state caused not only restriction of PRV infection in cell lines but also in an *in vivo* mouse model (Wang et al., 2020). Importantly, this sole study provides further evidence that BET BRDi can have diverse effects on virus infections via various means but also that they might be used as a future therapy.

## BET Bromodomain Inhibition and Betaherpesviruses

In contrast to alpha- and certainly gammaherpesviruses, little work has been published with use of BRDi during betaherpesvirus infection. In the absence of any investigation with roseolaviruses, HHV-6 and -7, observations have been made during lytic infection with the prototypic betaherpesvirsus, HCMV. HCMV maintains latency within CD14+ monocytes and CD34+ cells of the myeloid lineage, but only after primary infection and dissemination, which is thought to occur through productive infection of oral epithelial cells (Sinclair and Sissons, 2006). It is now clear that a number of viral genes are expressed during HCMV latency (Sinclair and Reeves, 2013) and maintenance of this phase takes place through epigenetic regulation and chromatin modification of the virus major immediate early promoter (MIEP) in order to repress the expression of the IE1/IE2 genes (Sinclair, 2010). Reactivation of the MIEP is usually associated with differentiation of cells to macrophage or dendritic cells (DCs) and the resulting changes to expression of host transcription factors. In turn, the production of the immediate early proteins IE72/IE86 leads to initiation of the usual cascade of herpesvirus gene expression, with early then late gene expression following during a full productive lytic cycle.

It is known that within the first 8 h of permissive infection with clinical isolates of HCMV, BRD4 along with CycT1 and CDK9 (the RNAPII-activating P-TEFb complex) accumulate at nuclear sites, known as the transcriptosome, where the transcriptionally active HCMV genome resides (Kapasi and Spector, 2008). BRD4 is able to recruit P-TEFb through its interaction with CycT1, however it has been reported that the presence of CDK9 at these transcriptosomes can be inhibited with CDK9 activity inhibitors (Kapasi and Spector, 2008). Hence, the direct relationship between these three proteins during HCMV is not fully understood. Regardless, the possibility that BRD4 may be recruiting the activating P-TEFb to the MIEP makes it an attractive target for inhibition, at least during lytic infection. In fact, work using the BRDi OTX015 (Noel et al., 2013) in a screen for anti-viral compounds, shown elsewhere to cause reactivation of HIV from latency (Lu et al., 2016), caused a decrease in the level of productive infection using an HCMV tegument protein pp28 GFP-tagged virus (Arend et al., 2017). Although this decrease in late protein expression could have been due to an initial restriction in transcription from the MIEP, the authors speculated that the effect may be due to an interference of the involvement of BRD2 in the replication of virus DNA, a necessary precursor to late herpesvirus gene expression (Arend et al., 2017).

The only other employment of BRDi during HCMV infection has shown little effect on the lytic cycle of the virus, albeit at only 2 h post-infection. The study by the Kristie group on BRDi-induced reactivation of HSV-1 also assessed the effect of JQ1, iBET-762 and HMBA on HCMV. However, in contrast to effects on HSV-1, little to no change in the level of immediate early (IE72) or early (UL44) transcripts was seen, consistent with the effects of BRD4 depletion with siRNAs (Alfonso-Dunn et al., 2017). Interestingly, though, reactivation of HSV-1 was shown to be dependent on the release of P-TEFb from repressive 7SK snRNP complexes in the cytoplasm (Bartholomeeusen et al., 2012), comparable to HIV studies elsewhere (Biglione et al., 2007), and the effect of BET BRDi on HCMV latently infected cells remains untested. Likewise, the possibility of BET BRDi employment for treatment of HCMV-associated cancers, where the virus has been reported to have an oncomodulatory role possibly through MYC dysregulation as seen with gammaherpesviruses (Herbein, 2018; Cobbs, 2019), has yet to be studied. However, given the varied effects of these inhibitors on both herpesvirus latent and lytically infected cells, as well as other human pathogenic viruses, these applications may be worthy of investigation.

## Discussion

The life cycles of many HHVs are known to be dependent on the activity of BET proteins at various stages throughout infection. Depending on the virus, BRD4 is integral for the control of HHV transcription (both latent and lytic), virus DNA replication and is associated, at least in part, with viral genome maintenance. Thus, small molecule inhibitors of BET proteins may make effective treatments at certain points during infection. Although BET BRDis cause HSV reactivation and augmentation of lytic infection, treatment of cells productively infected with EBV restricts viral lytic DNA replication, which may be common to treatment of HCMV lytic infection. However, the most promising effect of BET BRDi thus far has been its ability to control KSHV-infected tumor growth in mouse xenograft models. Here, restriction of host gene expression, such as *MYC*, limits cellular proliferation. However, it is presentation of viral lytic antigens after KSHV reactivation that allows infected cell targeting by pre-existing cytotoxic T cells. This scenario is akin to known “shock and kill” therapies for HIV patients where latency reversal agents (LRAs) have been trialed to varying efficiency to reduce latent HIV carriage (Thorlund et al., 2017; Ait-Ammar et al., 2019). However, to date, no treatment has shown complete removal of the latent HIV reservoir. While a curative treatment for viruses such as HIV would be highly beneficial, purging the latent reservoir of certain HHVs should prove advantageous to individuals undergoing, for example, stem cell or solid organ transplantation. Here, it has been proposed that the use of LRAs in HCMV seropositive donors and recipients prior to harvest and engraftment could allow purging of the latently infected cells and, as such, reduce reactivation event-related disease during immune suppression post-transplant (Poole et al., 2014; Wills et al., 2015). A pre-clinical study from our laboratory has shown the efficiency of using HDACi to induce HCMV lytic antigen production as a target for T cells (Krishna et al., 2016). With this in mind, we are now actively investigating the use of BET BRDi on latency/reactivation of HCMV as the basis for a refined “shock and kill” strategy to target the latent reservoir.

As a result of the success of BET BRDi use in the oncology setting, newer generations of BET-specific molecules are being designed. One approach is to target the protein of choice for proteasome-dependent degradation using Proteolysis Targeting Chimeras (PROTAC) (Cochran et al., 2019). Indeed, dBET1-targetting of BET proteins (Winter et al., 2015) showed similar increases of IE transcripts in HSV lytically infected cells when compared to pan-BET BRDi, such as JQ1 (Alfonso-Dunn et al., 2017). However, the effects of this style of molecule are not yet known during latency of the HHVs. A further strategy which may prove useful is the novel “block and lock” method, whereby small molecules are used to inhibit transcription from the viruses and lock promoters in a latent state (Nehme et al., 2019). Amongst various targets, a BRD4 BD1-specific compound (ZL0580) has recently shown the ability to “functionally cure” HIV by inhibiting Tat transactivation of the HIV promoter whilst causing chromatin-based repression (Niu et al., 2019; Vansant et al., 2020). Although this strategy might not allow targeting of infected cells to reduce latent carriage, the ability to inhibit reactivation of HHVs during treatment such as transplantation is alluring, whilst the possibility of inhibiting lytic infection in other clinical scenarios is particularly exciting. Be that as is may, further robust pre-clinical investigation will be necessary to confirm the potential of BET protein inhibitors as therapeutics for intervention of HHV-associated disease before human application.

## Author Contributions

IG wrote the manuscript and constructed the table/figure. MW and JS edited the manuscript. All authors contributed to the article and approved the submitted version.

## Conflict of Interest

The authors declare that the research was conducted in the absence of any commercial or financial relationships that could be construed as a potential conflict of interest.
